# Supplementary material for: Liberals and conservatives respond divergently to stereotype portrayals of race and gender
Source: Commun Psychol. 2025 Jul 17;3:109. doi: 10.1038/s44271-025-00287-6 (PMC12271372; doi:10.1038/s44271-025-00287-6)
Supplement: Supplementary file 2 — Supplementary Information [file 44271_2025_287_MOESM2_ESM.pdf]

Communications Psychology

Supplementary Materials and Information for Research Article: "Liberals and conservatives respond divergently to stereotype portrayals of racial minorities and women"

Authors: Elizabeth Q. Jiang & Margaret J. Shih

Supplementary Methods Appendix A:
Study Design Details and Sample Information

Study Description. We conducted one Pilot Study experiment, one Preliminary Study experiment, and four main Study quantitative experiments (Studies 1-4) using online samples of liberal and conservative participants. We report full methodological details for Studies 1-4 in the main text. In this Appendix A, we report detailed methods for Pilot Study and Preliminary Study.

Research Sample. The research sample consists of U.S. adults who are strongly and consistently liberal or conservative in their self-reported political ideology.

Sampling Strategy. We used convenience sampling via online survey panels (TurkPrime, Prolific, and CloudResearch Connect), given their ease and ability in reaching participants by ideology (and, in particular, difficult-to-reach Conservative participants).

A note on recruiting samples of politically liberal and politically conservative participants using online panels: The online survey panels (e.g., TurkPrime and Prolific) feature pre-screening tools based on participants' demographics. We used the panels' pre-screening questions capturing participants' political ideology in attempt to recruit balanced samples of liberals and conservatives. We do not use the online panels' pre-screening tools alone to identify participants' political ideology in our analyses, which may be outdated; to identify participants' political ideology, we use our demographic questions, which are asked after the main dependent variable measures and at the end of each survey. Our online surveys were advertised in terms of general perceptions, attitudes, behavior, and decision-making, and in no way mentioned political ideology, political beliefs, stereotypes, race, or gender. Additionally, researchers' usage of the pre-screening tool provided by the online survey panel is not shared with participants. We expect no priming effect of political ideology on our results.

Data Collection. Data was collected online using online survey panels, TurkPrime, Prolific, or CloudResearch Connect. Participants were online workers who would complete an online experimental survey, which was hosted on Qualtrics.

Regarding sample size calculations:

For the Pilot Study and for Preliminary Study, we used G*Power to calculate the required sample size needed given effect size, error probability, number of groups, and statistical power inputs (please see preregistration documents for more details, including a screenshot of our G*Power inputs). On top of the observations required by the G*Power calculation, we added additional participants based on an assumed exclusion rate due to preregistered data exclusions or data quality concerns (e.g., 20%; please see Data exclusions section below for more details).

Timing. Timing for each study is as follows:

Pilot Study: September 30, 2022 and October 5-6, 2022.

Preliminary Study: January 31, 2023 (8:00am) – January 31, 2023 (11:20am).

Data Exclusions. Data exclusions are listed below:

Pilot Study: As preregistered, we excluded data from participants who did any of the following: did not complete the full survey including the final question (n = 92), were duplicate respondents (n = 2), and did not respond correctly to attention check questions (n = 1) including an additional participant who answered their demographic age question as being under 18 years old, which we believe was a typo on the participant's part (n = 1). In total, this led to excluding 96 participants and yielded a final sample of n = 325 participants. Please note, the n = 325 includes random assignment of 115 participants to the stereotype congruent condition, 105 participants to the stereotype incongruent condition, and 105 participants to the stereotype neutral condition. We do not report the descriptive or inferential results of the stereotype neutral condition, as we do not use these artefacts or condition design in future studies. This yields a Pilot Study sample size of n = 220.

Preliminary Study: As preregistered, we excluded data from participants who did any of the following: did not complete the full survey including the final question (more details below in the Non-Participation section, n = 74), were duplicate respondents (n = 0), or who did not respond correctly to attention check questions (n = 7). In total, this led to excluding data from 81 participants. Additionally, we excluded from analysis data from individuals who were not consistently and strongly politically self-identified (i.e., excluded participants who were politically moderate or politically independent, n = 100; more details in our Main Manuscript and Supplementary Materials). This yielded a final sample of n = 196 Liberal and Conservative participants.

Non-Participation. Non-participation is listed below:

Pilot Study: 92 participants did not complete the full survey including the final question. We received raw data from *n* = 421 participants, yielding a 78.1% completion rate.

Preliminary Study: 74 participants did not complete the full survey including the final question. We received raw data from *n* = 377 participants, yielding a 80.4% completion rate.

Sample Demographics.

Please see the following pages for participant race and gender breakdowns for each study (Supplementary Tables S1-S2).

Supplementary Table S1. Pilot Study: Participant Race and Gender

Participant gender	Participant race	#	% of sample
Male	White	87	39.5%
	Black	11	5.0%
	Asian	6	2.7%
	Hispanic	10	4.5%
	Native Hawaiian or Pacific Islander	1	0.5%
	Other	2	0.9%

Female	White	86	39.1%
	Black	7	3.2%
	Asian	1	0.5%
	Hispanic	2	0.9%
	American Indian or Alaska Native	2	0.9%
	Other	3	1.4%
Other	White	1	0.5%
	Other	1	0.5%

Supplementary Table S2. Preliminary Study: Participant Race and Gender

Participant gender	Participant race	#	% of sample
Male	White	52	26.50%
	Black	13	6.60%
	Asian	5	2.60%
	Hispanic	8	4.10%
	Native Hawaiian or Pacific Islander	1	0.50%
	Other	1	0.50%
Female	White	98	50%
	Black	8	4.10%
	Asian	3	1.50%
	Hispanic	5	2.60%
	Other	1	0.50%
Other	American Indian or Alaska Native	1	0.50%

Supplementary Methods Appendix B:
Supplementary Results

Pilot Study: Testing Advertisement Stimuli

The preregistered pilot study (n = 220) measured perceived **realism** of our custom ads using an index consisting of 5 items (e.g., "I have seen advertisements just like these in the real world" and "The ads I saw look like fake ads (R).") (a = .82). Out of a Likert 1-7 scale wherein 1=strongly disagree, 4=neutral/neither, 7=strongly agree, we found that the *stereotype congruent condition* participants perceived the ads to be very realistic (M = 5.87, SD = 1.01), while the *stereotype incongruent condition* perceived the ads to be relatively less realistic (M = 5.51, SD = 1.03), although both are still significantly above the neutral point of 4 (congruent ads: $t(114)$ = 19.82, 95% CI [5.68, 6.06], $p < .001$; incongruent ads: $t(104)$ = 15.06, 95% CI [5.31, 5.71], $p < .001$). Further, we measured **stereotype alignment** of the ads using an index of 5 items (e.g., "These advertisements illustrate general stereotypes that are commonly held by society" and "The way the models are depicted in the ads does not align with common stereotypes (R).") (a = .86). Out of a Likert 1-7 scale wherein 1=strongly disagree, 4=neutral/neither, 7=strongly agree, we found that the *stereotype congruent condition* participants perceived the ads to be congruent with commonly held stereotypes (M = 5.07, SD = 1.15), while the *stereotype incongruent participants* perceived the ads to be incongruent (M = 2.81, SD = 1.19). Additionally, each of these means were significantly different from the neutral point of 4 in their relative directions (congruent ads: $t(114)$ = 9.90, 95% CI [4.85, 5.28], $p < .001$; incongruent ads: $t(104)$ = -10.28, 95% CI [2.58, 3.04], $p < .001$).

Preliminary Study: Testing Attitudes

We first conducted a preliminary experiment ("Preliminary Study") with U.S. citizens who were randomly assigned to stereotype congruent vs. incongruent conditions that depicted racial minorities and manipulated race and gender stereotypes in workplace advertisements. We asked participants to rate their liking, comfort with, and persuasiveness of the advertisement, which we collapsed into an index measure of **attitudinal perceptions** (see Supplementary Materials Appendix B subsection "Index Measures" for more details on individual items and index outcomes).

Following our pre-registration, we first conducted a one-way ANOVA to test for a main effect of stereotype portrayal on attitudes and found no significant effect (M_{cong} = 5.24, SD_{cong} = 1.02, M_{incong} = 5.24, SD_{incong} = 0.96; $F_{(1, 194)} < 0.01$, p = .952, $\eta^2_p < .01$, 95% CI = [0.00, 1.00]). We find no support for our prediction that participants will prefer stereotype congruent portrayals more than stereotype incongruent portrayals.

Next, we ran a factorial ANOVA testing for an interaction effect of stereotype portrayal and political ideology on attitudes. Our results show a significant interaction effect ($M_{\text{cons*cong}}$ = 5.56, $SD_{\text{cons*cong}}$ = 1.15, $M_{\text{cons*incong}}$ = 5.03, $SD_{\text{cons*incong}}$ = 1.42, $M_{\text{libs*cong}}$ = 5.12, $SD_{\text{libs*congruent}}$ = 0.94, $M_{\text{libs*incong}}$ = 5.34, $SD_{\text{libs*incong}}$ = 0.59; $F_{(1, 192)}$ = 6.32, p = .013, η^2_p = .03, 95% CI = [0.00, 1.00]). These results were robust in exploratory linear regressions with and without holding constant participants' age, race, gender, income, and educational attainment (b = 0.75, SE = 0.30, p = .013, η^2_p = .03, 95% CI = [0.00, 1.00]; b = 0.76, SE = 0.30, p = .013, η^2_p = .03, 95% CI = [0.00, 1.00]). Conducting an exploratory post-hoc Tukey's Honest Significant Differences (Tukey HSD) multiple pair-wise difference test, we found no significant pairwise differences, suggesting that our sample

size is limited or the interaction effect is small despite being significant at $p < .05$. We address sample size limitations in our main studies, Studies 1-4. This interaction effect provides support for our prediction that political ideology will moderate attitudinal ratings of the stereotype congruent and incongruent ads.

Finally, we conducted a one-way ANOVA to test for a main effect of stereotype portrayal on time spent viewing the target advertisements, having predicted that stereotype incongruent ads will be more surprising (i.e., will be viewed for longer) than stereotype congruent ads. We found no differences in time spent (in seconds) viewing target advertisments between stereotype portrayal conditions (M_{cong} = 693, SD_{cong} = 256, M_{incong} = 650, SD_{incong} = 280; $F_{(1, 194)} = 1.25$, $p = .264$, $\eta^2_p < .01$, 95% CI = [0.00, 1.00]), thus failing to provide support for this prediction.

Index Measures

Political Ideology

We specified our liberal and conservative political ideology categories using a sociopolitical index score, which was the composite of four items: political leaning (“In general, how liberal (left-wing) or conservative (right-wing) are you?”), political party (“Which of the following do you most closely identify as?”), fiscal ideology (“When it comes to economic/fiscal issues and policies, which of the following do you most closely identify as?”), and social ideology (“When it comes to social issues and policies, which of the following do you most closely identify as?”) (*a* >= .98, Studies 1-4) (Likert 1-7: 1 = Very liberal/Strongly Democrat, 4 = Moderate/Middle-of-the-road/Independent, 7 = Very conservative/Strongly Republican). We sought participants with strong and consistent sociopolitical ideology orientations and self-identifications—as such, we categorized respondents who had a sociopolitical index score between 1 = “very liberal” and 2 = “liberal” as liberal, while those who had an index score between 6 = “conservative” and 7 = “very conservative” were categorized as conservative; respondents who did not meet these political ideology categorizations (i.e., politically moderate or independent participants) were not included in analyses. Further, our preregistered hypotheses and analyses focus on the difference between liberals and conservatives, and thus we exclude from analysis any politically moderate or independent participants.

Attitudinal Preferences

Preferences are captured in an attitudes index, which consisted of liking (e.g., “In general/overall, how much did you like the ads you saw?” and “I really did not like the images of the ads” (reverse-coded)), comfort (e.g., “How comfortable or uncomfortable were you with the ads?” and “The ads made me feel uneasy” (reverse-coded)), and persuasiveness (e.g., “How would you describe your desire to own or receive the products/services featured in the ads?” and “I want to experience or purchase these products/services”) items (Likert 1-7; 5 items, *a* = .81, Preliminary Study; 16 items, *a* >= .92, Studies 1-4). A list of all items used in Studies 1-4 is below:

Supplementary Table S3: Attitudinal Preferences Items.

Construct	Item Text

Liking	To what extent do you like the *products* featured in the advertisement?
	To what extent do you like the *models* featured in the advertisement?
	In general/overall, how much did you like the ads you saw?
	I really did not like the images of the ads (reverse-coded).
	There was something about the ads that I just didn't like (reverse-coded).
	I thought the ads were great and I really liked them.
Comfort	How would you describe your comfort with the ads? In other words, how comfortable or uncomfortable were you with the ads?
	Looking at the ads, I felt perfectly at ease and comfortable.
	I noticed I felt rather tense and uncomfortable when looking at the ads (reverse-coded).
	I enjoyed viewing the ads.
	The ads made me feel uneasy (reverse-coded)
Persuasiveness	How would you describe your ***desire to own or receive the products/services*** featured in the ads?
	I want to experience or purchase these products/services.
	I did not really take a fancy to any of these products and services (reverse-coded).
	The ads showed products and services I actually want and would like to have in my everyday life.
	Looking at the ads, I did not really want to own any of them (reverse-coded).

Note. All items were measured on a Likert scale 1-7 with scale labels based on question (1 = Very much dislike/uncomfortable/no desire at all, 7 = Very much like/comfortable/desire; 1 = Strongly disagree, 4 = Neutral, 7 = Strongly agree).

Economic Perceptions

Economic perceptions of monetary value are captured in an index, which consisted of a willingness to pay item ("Thinking about yourself… In total, how much would you be willing to pay for all of these products/services (in U.S. dollars $)?" (Sliding bar from $0 to $500)") and a worth to others item ("In total, how much do you think all of these products/services are worth to the average American (in terms of U.S. dollars $)?" (Sliding bar from $0 to $500)") ($a >= .78$, measured in Studies 1-4).

Study 1 Supplementary Results

Secondary Results

Following our preregistration and to replicate Preliminary Study, we conduct a one-way ANOVA testing for a main effect of stereotype portrayal on attitudes. As in

Preliminary Study, we find no significant differences in attitudes between stereotype portrayal conditions (M_{cong} = 5.02, SD_{cong} = 1.00, M_{incong} = 5.09, SD_{incong} = 0.98; $F_{(1, 956)}$ = 1.32, p = .252) and fail to support our prediction that participants would overall favor stereotype congruent portrayals over stereotype incongruent portrayals.

We also conduct a confirmatory one-way ANOVA testing for a main effect of stereotype portrayal on downstream hiring, having predicted a significant effect. We found no significant differences in downstream hiring selections between stereotype conditions (M_{cong} = 0.31, SD_{cong} = 0.46, M_{incong} = 0.30, SD_{incong} = 0.46; $F_{(1, 956)}$ = 0.04, p = .842), thus failing to support this prediction.

$p_{augmented}$ *Results for Main Analyses*

In Study 1, we conducted two pre-registered waves of participant recruitment using a mini meta-analysis approach in merging the data (Goh et al., 2016). Following Sagarin et al.'s guidance regarding sample augmentation (2014), we calculate and report $p_{augmented}$ for Study 1's results below.

Attitudes. After running the first wave of participants (Study 1 n_{Wave1} = 684), we found no significant interaction effect of political ideology and stereotype portrayal on attitudes ($F_{(1, 680)}$ = 3.24, p = .072, η^2_p < .01). Please note n_{Wave1} did not meet our *a priori* desired sample of 800 participants. We conducted a preregistered second wave of recruitment to increase the number of politically conservative and politically liberal participants and to achieve our desired sample size. With Wave 1 and Wave 2 (n_{Wave2} = 274), we obtained a full Study 1 sample of n = 958. For the full sample, the interaction effect was significant ($F_{(1,954)}$ = 3.98, p = .046, η^2_p < .01). Following Sagarin et al.'s guidance (2014), we calculated $p_{augmented}$ for the full sample and found $p_{augmented}$ = [.055, .072]; Sagarin et al. note that $p_{augmented}$ range will always exceed .05.

Economic Perceptions. For the first wave of participants, we found a significant interaction effect of political ideology and stereotype portrayal on economic perceptions ($F_{(1, 680)}$ = 8.45, p = .004, η^2_p = .01). For the full sample, the interaction effect remained significant ($F_{(1, 954)}$ = 6.23, p = .013, $\eta^2 p$ < .01). We calculated $p_{augmented}$ for the full sample and found $p_{augmented}$ = [.050, .053].

Downstream Hiring. Running the first wave of participants, we found no significant interaction effect of political ideology and stereotype portrayal on downstream hiring ($F_{(1, 680)}$ < 0.01, p = .99). For the full sample, the interaction effect was not significant ($F_{(1,954)}$ < 0.01, p = .98).

Following our preregistration, we test for a significant main effect by political ideology and found significant effect in our Wave 1 sample ($t_{(682)}$ = 5.16, p < .001, Cohen's d = .40) and in our full sample ($t_{(956)}$ = 5.39, p < .001, Cohen's d = .35). We calculated $p_{augmented}$ for the full sample and found $p_{augmented}$ = [.050, .050].

Exploratory Linear Regression including Political Moderates

To examine the relationship between stereotype congruence, attitudes, and the full spectrum of political ideology, we conducted an exploratory multiple regression analysis that incorporated the previously excluded political moderates into our political ideology categories. We specified political ideology (conservative [reference group], liberal, or

moderate), stereotype condition (congruent [reference group] or incongruent), and their interaction as predictors of our attitudinal perceptions outcome.

Similar to our main results, we found a significant interaction effect of stereotype portrayal and political ideology on attitudinal perceptions ($M_{cons*cong}$ = 4.91, $M_{cons*incong}$ = 4.85, $M_{libs*cong}$ = 5.10, $M_{libs*incong}$ = 5.29, $M_{mods*cong}$ = 5.11, $M_{mods*incong}$ = 5.02; *b* = 0.25, *SE* = 0.13, *p* = .046). In post-hoc comparisons, we found significant pairwise differences between conservatives and liberals in the incongruent stereotype portrayal conditions ($t_{(1133)}$ = 5.00, *p* <.001, *d* = 0.46) and between conservatives evaluating congruent portrayals and liberals evaluating incongruent portrayals ($t_{(1133)}$ = 4.25, *p* <.001, *d* = 0.40). All other pairwise comparisons were not significant (*p*'s > .05).

Study 2 Supplementary Results

Mediation Results

In Study 2, we consider why perceptions diverged on the basis of political ideology and stereotype portrayal. We collected additional measures on expectations and norms (see Supplementary Table S4 for items). These measures were administered in the procedures of Study 2 immediately following the advertisements and prior to our dependent measures. Running 5,000 bootstrapped samples using the standard PROCESS Model 8 script in R (Hayes, 2017), we fitted a moderated-mediation model specifying stereotype portrayal as the independent variable, political ideology as the moderator, expectations as the mediator, and attitudinal perceptions as the outcome variable.

We found that stereotype portrayal was a positive predictor of expectations (*b* = 0.15, *SE* = 0.06, *t* = 2.35, *p* = .02) but not attitudinal perceptions (*p* = .42), suggesting full mediation. Expectations (*b* = 1.00, *SE* = 0.03, *t* = 30.51, *p* < .001) was a positive predictor of attitudinal perceptions. Political ideology was a negative moderator of the link between stereotype portrayal and expectations (*b* = -0.23, *SE* = 0.10, *t* = -2.28, *p* = .02), but not of the link between stereotype portrayal and attitudinal perceptions (*p* = .46). In support of a moderated mediation, there was a significant indirect effect of stereotype portrayal through expectations on attitudinal perceptions conditional on political ideology (index = -0.23, *SE* = 0.11, CI [-0.44; -0.03]), driven by a significant indirect effect among liberals (*b* = 0.15, *SE* = 0.06, CI [0.03, 0.27]) rather than among conservatives (*b* = -0.08, *SE* = 0.09, CI [-0.25, 0.09]). Figure S1 illustrates the moderated mediation model.

The moderated mediation also held when specifying economic perceptions as the outcome variable. Stereotype portrayal was a positive predictor of expectations, but not economic perceptions (*p* = .17). Expectations (*b* = 36.62, *SE* = 5.44, *t* = 6.73, *p* < .001) was a positive predictor of economic perceptions. Political ideology was a negative moderator of the link between stereotype portrayal and expectations, but not of the link between stereotype portrayal and economic perceptions (*b* = -3.84, *SE* = 16.48, *t* = -0.23, *p* = .82). Further, there was significant indirect effect of stereotype portrayal through expectations on economic perceptions at different levels of political ideology (index = -8.41, *SE* = 4.06, CI [-17.20; -1.05]), with a significant indirect effect among liberals (index = 5.43, *SE* = 2.25, CI [1.32; 10.15]) and a null indirect effect among conservatives (index = -2.98, *SE* = 3.31, CI [-9.74; 3.23]) (see Figure S1).

Finally, when specifying behavioral selection as the outcome variable, the moderated mediation did not hold, as stereotype portrayal was a positive predictor of both expectations and behavioral selection (*b* = -0.42, *SE* = 0.20, *Z* = -2.12, *p* = .03). The index of moderated mediation also showed null significance (index = -0.03, *SE* = 0.03, CI [-0.09; 0.02]).

Supplementary Figure S1. Moderated Mediation Model for Study 2 and Study 3.

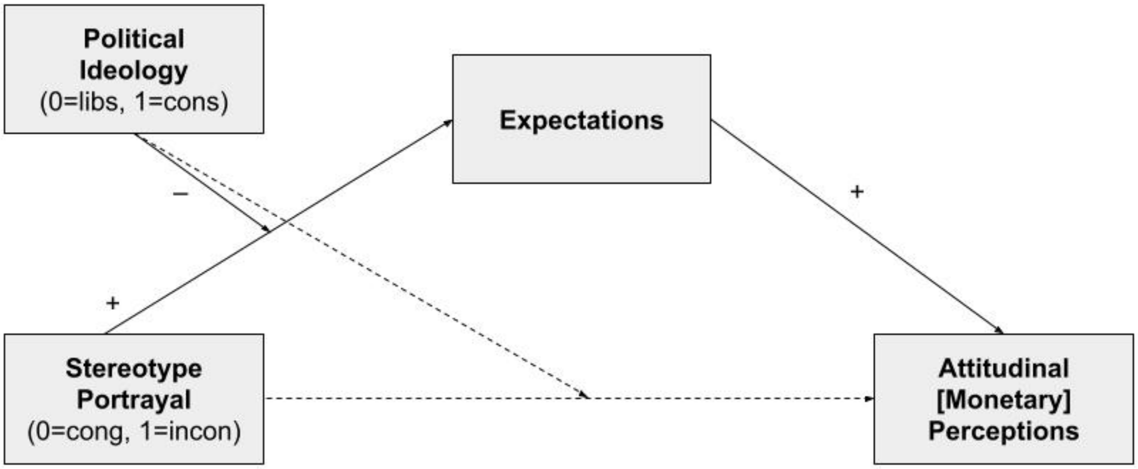

Expectation Index

Expectations Mediator. Expectations are captured in an index, which consisted of items such as "I did not find anything strange about the ads" and "I found the ads to be different from what I expected" (reverse-coded) (Likert 1-7; 22 items, *a* >= .85, measured in Studies 2-3). A list of all items is below in Supplementary Table S4:

Supplementary Table S4: Expectation Index Question Items.

**Question Stem**	**Item Text**
Thinking about the advertisement images you saw (the models and the products/services), please answer the following questions:	The images were what you would expect most ads to look like.
	There was something unusual about the ads (reverse-coded).
	I found the ads to be different from what I expected (reverse-coded).
	I did not find anything strange about the ads.
	I liked the images and ads I just saw.
	The images I viewed were pleasant to me.
	I found the images and ads to be unpleasant (reverse-coded).
	What I just viewed was undesirable (reverse-coded).
	The ads made me feel a positive way.
	The ads made me feel negative (reverse-coded).
	…were completely expected.
	…were completely unexpected (reverse-coded).

These advertisement images (the models and the products/services)…	…surprised me only very slightly.
	…surprised me a great deal (reverse-coded).
	…were only very slightly unexpected.
	…were only very slightly expected (reverse-coded).

Note. All items were measured in Studies 3-4. All items were measured on a Likert scale 1-7 (1 = Strongly disagree, 4 = Neutral, 7 = Strongly agree).

Exploratory Linear Regression including Political Moderates

We conducted an exploratory multiple regression analysis that incorporated political moderates into our political ideology categories. We specified political ideology (conservative [reference group], liberal, or moderate), stereotype condition (congruent [reference group] or incongruent), and their interaction as predictors of attitudinal perceptions.

We found a significant interaction effect of stereotype portrayal and political ideology on attitudinal perceptions ($M_{cons*cong}$ = 4.90, $M_{cons*incong}$ = 4.79, $M_{libs*cong}$ = 5.10, $M_{libs*incong}$ = 5.29, $M_{mods*cong}$ = 4.92, $M_{mods*incong}$ = 5.19; *b* = 0.30, *SE* = 0.14, *p* = .031). Post-hoc comparisons revealed significant pairwise differences between conservatives and liberals in the incongruent stereotype portrayal conditions ($t_{(1038)}$ = 5.10, $p < .001$, *d* = 0.49), between conservatives evaluating congruent portrayals and liberals evaluating incongruent portrayals ($t_{(1038)}$ = 3.96, *p* = .001, *d* = 0.38), and between liberals evaluating congruent portrayals and conservatives evaluating incongruent portrayals ($t_{(1038)}$ = 3.05, *p* = .029, *d* = 0.29). All other pairwise comparisons were not significant (*p*'s > .05). These exploratory results are in line with our main results showing a significant interaction.

Study 3 Supplementary Results

Mediation Results

We replicated our moderated mediation model for attitudinal perceptions from Study 2. Stereotype portrayal was a positive predictor of expectations (*b* = 0.37, *SE* = 0.08, *t* = 4.96, $p < .001$) and of attitudinal perceptions (*b* = 0.24, *SE* = 0.07, *t* = 3.25, *p* = .001). Expectations (*b* = 0.90, *SE* = 0.04, *t* = 25.64, $p < .001$) was a positive predictor of attitudinal perceptions. Political ideology was a negative moderator of the link between stereotype portrayal and expectations (*b* = -0.81, *SE* = 0.11, *t* = -7.40, $p < .001$), but not of the link between stereotype portrayal and attitudinal perceptions (*p* = .52). In further support of a moderated mediation, there was a significant indirect effect of stereotype portrayal through expectations on attitudinal perceptions conditional on political ideology index = -0.72, *SE* = 0.10, CI [-0.92; -0.53]), driven by a significant indirect effect among both liberals (*b* = 0.34, *SE* = 0.06, CI [0.21, 0.26]) and conservatives (*b* = -0.39, *SE* = 0.08, CI [-0.54, -0.24]). Figure S1 illustrates the moderated mediation model. We found no significant moderated mediation for economic perceptions or for behavioral selections.

Exploratory Linear Regression including Political Moderates

We conducted an exploratory multiple regression analysis that incorporated political moderates into our political ideology categories, specifying political ideology (conservative [reference group], liberal, or moderate), stereotype condition (congruent

[reference group] or incongruent), and their interaction as predictors of attitudinal perceptions.

We found a significant interaction effect of stereotype portrayal and political ideology on attitudinal perceptions ($M_{cons*cong}$ = 5.10, $M_{cons*incong}$ = 4.89, $M_{libs*cong}$ = 4.24, $M_{libs*incong}$ = 4.82, $M_{mods*cong}$ = 4.62, $M_{mods*incong}$ = 4.75; b = 0.79, SE = 0.14, p < .001). Post-hoc comparisons revealed significant pairwise differences between conservatives and liberals in the congruent stereotype portrayal conditions ($t_{(885)}$ = 8.37, p < .001, d = 0.86), between liberals evaluating congruent portrayals and conservatives evaluating incongruent portrayals ($t_{(885)}$ = 6.36, p < .001, d = 0.65), and between liberals evaluating congruent versus incongruent stereotype portrayals ($t_{(885)}$ = 5.81, p < .001, d = 0.58). Two pairwise comparisons with political moderates were significant: conservatives expressed significantly higher attitudes than moderates evaluating congruent portrayals ($t_{(885)}$ = 3.08, p = .026, d = 0.32), and moderates evaluating incongruent portrayals expressed significantly higher attitudes than liberals evaluating congruent portrayals ($t_{(885)}$ = 3.34, p = .011, d = 0.34). All other pairwise comparisons were not significant (p's > .05). These exploratory results are in line with the significant interaction result from our main results.

Study 4 Supplementary Results

Pilot Testing Advertisement Stimuli for Study 4

A second pilot study (n = 141) was conducted to test the experimental stimuli we plan to use in Study 4. This new set of experimental stimuli were selected to enhance similarity in model facial expression, body language, and environment across conditions (see Table 1 for experimental stimuli).

As in our previous pilot study, we measured perceived **realism** of our custom ads using an index consisting of 5 items (e.g., "I have seen advertisements just like these in the real world" and "The ads I saw look like fake ads (R).") (a = .85) (Likert 1-7 scale wherein 1=strongly disagree, 4=neutral/neither, 7=strongly agree). As we expected, all ads, regardless of stereotype portrayal and model race, were seen to be highly realistic (M = 5.41, SD = 1.21) and to have a realism rating greater than the mid-point of the scale (t(140) = 13.84, 95% CI [5.21, 5.61], p < .001). Looking next at stereotype portrayal, we also found that the *stereotype congruent condition* participants perceived the ads to be very realistic (M = 5.65, SD = 1.12), while the *stereotype incongruent condition* perceived the ads to be relatively less realistic (M = 5.22, SD = 1.26), although both are still significantly above the neutral point of 4 (congruent ads: t(63) = 11.79, 95% CI [5.37, 5.93], p < .001; incongruent ads: t(76) = 8.48, 95% CI [4.93, 5.50], p < .001). Then, looking between the conditions varying model race featured in the advertisments, we found that the *minority model condition* participants perceived the ads to be very realistic (M = 5.45, SD = 1.19), while the *white model condition* participants perceived the ads to be relatively less realistic (M = 5.38, SD = 1.24), although both are still significantly above the neutral point of 4 (minority models: t(68) = 10.14, 95% CI [5.16, 5.73], p < .001; white models: t(71) = 9.40, 95% CI [5.08, 5.67], p < .001). Finally, looking at the interaction between stereotype portrayal and model race on realism ratings, we found no significant interaction effect (F(1, 137) = 1.85, p = .176). Overall, our pilot study results suggest that participants did not detect any differences in realism of the advertisements across conditions and that they rated all ads as highly realistic.

Further, we measured **stereotype alignment** of the ads using an index of 5 items (e.g., “These advertisements illustrate general stereotypes that are commonly held by society” and “The way the models are depicted in the ads does not align with common stereotypes (R).”) (a = .92) (Likert 1-7 scale wherein 1=strongly disagree, 4=neutral/neither, 7=strongly agree). As we had expected, we found that the *stereotype congruent condition* participants perceived the ads to be congruent with commonly held stereotypes (M = 5.21, SD = 1.27), while the *stereotype incongruent participants* perceived the ads to be incongruent (M = 2.98, SD = 1.35). Additionally, each of these means were significantly different from the neutral point of 4 in their relative directions (congruent ads: $t(63) = 7.62$, 95% CI [4.89, 5.53], $p < .001$; incongruent ads: $t(76) = -6.59$, 95% CI [2.68, 3.29], $p < .001$). Overall, the experimental stimuli tested in this pilot study have stereotype alignment ratings that match the stereotype portrayal condition manipulation.

Exploratory Two-Way Factorial ANOVAs Specifying Stereotype Portrayal x Model Race

We ran exploratory factorial ANOVAs of *stereotype portrayal* and *model race* on our outcome measures to probe whether any difference in attitudes, economic perceptions, or downstream hiring existed due to stereotype congruency and race of advertisement models. We had no *a priori* hypotheses regarding the effect of these two factors' interaction on our outcome measures and had not preregistered these analyses as confirmatory analyses.

We first tested for an interaction effect of stereotype portrayal and model race on attitudes. We found no significant interaction effect ($M_{cong*minor}$ = 4.82, $SD_{cong*minor}$ = 1.04, $M_{cong*white}$ = 4.58, $SD_{cong*white}$ = 1.06, $M_{incong*minor}$ = 4.82, $SD_{incong*minor}$ = 1.03, $M_{incong*white}$ = 4.66, $SD_{incong*white}$ = 0.99; $F_{(1, 2480)}$ = 0.91, p = .341). Evaluating main effects, we also found no significant main effect of stereotype condition on attitudes (M_{cong} = 4.70, SD_{cong} = 1.06, M_{incong} = 4.74, SD_{incong} = 1.01; $F_{(1, 2480)}$ = 0.95, p = .330). We did find a significant main effect of model race on attitudes (M_{minor} = 4.82, SD_{minor} = 1.03, M_{white} = 4.62, SD_{white} = 1.03; $F_{(1, 2480)}$ = 23.76, $p < .001$, $\eta^2_p < .01$), revealing that, overall, participants assigned to view minority models liked the target advertisements more than participants assigned to view white models regardless of stereotype congruency.

Next, we tested for an interaction effect on economic perceptions and found no significant interaction effect of stereotype portrayal and model race on our monetary outcomes ($M_{cong*minor}$ = \$183, $SD_{cong*minor}$ = \$120, $M_{cong*white}$ = \$182, $SD_{cong*white}$ = \$115, $M_{incong*minor}$ = \$187, $SD_{incong*minor}$ = \$123, $M_{incong*white}$ = \$187, $SD_{incong*white}$ = \$118; $F_{(1, 2480)}$ = 0.03, p = .855). We also found no significant main effect of stereotype portrayal (M_{cong} = \$183, SD_{cong} = \$118, M_{incong} = \$187, SD_{incong} = \$120; $F_{(1, 2480)}$ = 0.87, p = .352) or of model race (M_{minor} = \$185, SD_{minor} = \$121, M_{white} = \$185, SD_{white} = \$117; $F_{(1, 2480)}$ = 0.01, p = .916) on economic perceptions.

Finally, we tested for an interaction effect of stereotype portrayal and model race on downstream hiring and found no significant interaction effect ($M_{cong*minor}$ = 0.55, $SD_{cong*minor}$ = 0.5, $M_{cong*white}$ = 0.52, $SD_{cong*white}$ = 0.5, $M_{incong*minor}$ = 0.48, $SD_{incong*minor}$ = 0.5, $M_{incong*white}$ = 0.50, $SD_{incong*white}$ = 0.5; $F_{(1, 2480)}$ = 1.15, p = .284). In terms of main effects, we found that stereotype portrayal (M_{cong} = 0.53, SD_{cong} = 0.5, M_{incong} = 0.49, SD_{incong} = 0.5; $F_{(1, 2480)}$ = 5.02, p = .025, $\eta^2_p < .01$), but not model race (M_{minor} = 0.51, SD_{minor} = 0.5, M_{white} = 0.51, SD_{white} = 0.5; $F_{(1, 2480)}$ = 0.04, p = .849), was a significant standalone

predictor of downstream hiring, suggesting that participants assigned to the stereotype congruent conditions were more likely than those assigned to the stereotype incongruent conditions to hire a stereotype congruent job candidate regardless of model race.

Vignette Hiring Stimuli (Studies 1-3)

To measure downstream behavioral choices, we included a vignette-style question. The scenario asked participants to hire one I.T./software candidate out of six demographically unique options (see Figure S2). We used dummy coding for these measures. Participants who selected the Asian male candidate for software were coded as making a congruent selection, and participants who selected any other software candidate were coded as making an incongruent selection (0=incongruent candidate selected, 1=congruent candidate selected). The vignette candidates' names and headshots were pre-tested for equivalent competence ratings and accurate race and gender portrayals. In the pre-test, we conducted a pilot test on perceptions of the vignette candidates using a random assignment between-conditions design (n = 138). We found no differences in competence ratings ("How would you rate [Name]/s competence?"; Likert 1 = Not at all competent, 7 = Extremely competent) between twelve different candidate profiles (M_{AF1} = 5.50, M_{AF2} = 5.78, M_{AM1} = 5.00, M_{AM2} = 5.36, M_{BF1} = 5.55, M_{BF2} = 5.33, M_{BM1} = 5.67, M_{BM2} = 5.58, M_{WF1} = 5.55, M_{WF2} = 4.83, M_{WM1} = 5.50, M_{WM2} = 4.38; p = .32). We selected six demographically different candidate profiles for the software vignette measure.

Supplementary Figure S2. Vignette Hiring Stimuli.

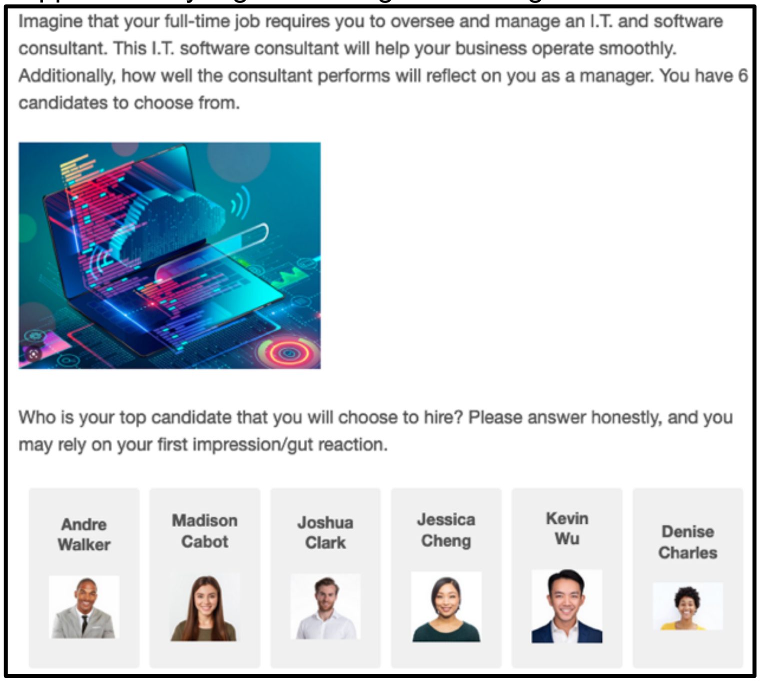

Vignette Hiring Stimuli (Study 4)

To measure downstream behavioral choices, we included a vignette-style question. The scenario asked participants to hire one I.T./software candidate out of two demographically unique options. We used dummy coding for these measures. Participants who selected the Asian male candidate for software were coded as making

a congruent selection, and participants who selected the Black female software candidate were coded as making an incongruent selection (0=incongruent candidate selected, 1=congruent candidate selected). The Asian male and Black female candidates' names and headshots were maintained from a previous pre-test.

Supplementary References

Goh, J. X., Hall, J. A., & Rosenthal, R. (2016). Mini meta-analysis of your own studies: Some arguments on why and a primer on how. *Social and Personality Psychology Compass*, *10*(10), 535-549. https://doi.org/10.1111/spc3.12267

Hayes, Andrew F. (2017), *Introduction to Mediation, Moderation, and Conditional Process Analysis: A Regression-Based Approach*, New York, NY: Guilford.

Sagarin, B. J., Ambler, J. K., & Lee, E. M. (2014). An ethical approach to peeking at data. *Perspectives on Psychological Science*, *9*(3), 293-30. https://doi.org/10.1177/174569161452821
